# Tibial Mechanical Axis Is Nonorthogonal to the Floor in Varus Knee Alignment

**DOI:** 10.1016/j.artd.2021.03.009

**Published:** 2021-04-15

**Authors:** Stefano A. Bini, Christopher C. Chung, Scott A. Wu, Erik N. Hansen

**Affiliations:** a Department of Orthopaedic Surgery, University of California, San Francisco, San Francisco, CA, USA; b University of Virginia School of Medicine, Charlottesville, VA, USA; c Northwestern University Feinberg School of Medicine, Chicago, IL, USA

**Keywords:** Tibial axis orientation angle, Mechanical alignment, Total knee arthroplasty, Tibial mechanical axis, Knee angles

## Abstract

**Background:**

Classical models of the knee assume the joint line is parallel to the floor and the tibial mechanical axis (TMA) is orthogonal to the floor. Our study characterizes the angle subtended by the TMA and floor during bipedal stance, called the tibial axis orientation angle (TAOA), and tests the assumption that the TMA should be orthogonal to the floor.

**Methods:**

We reviewed the nonoperative knee on full-length, standing radiographs in patients undergoing total knee arthroplasty between 2013 and 2017. Radiographic measurements were obtained for hip-knee-ankle axis, medial proximal tibial angle (MPTA), joint line orientation angle, and TAOA and correlated by regression analysis. The cohort was stratified by hip-knee-ankle axis alignment to determine statistical differences in knee angle values. Demographic data were collected to assess associations with knee angles.

**Results:**

Our cohort included 68 patients, with 56% female and average age of 62.3 years. Varus knees comprised 56% of the cohort, with 7% neutral and 37% valgus. The cohort demonstrated an MPTA of 3.06°, TAOA of 2.67°, and joint line orientation angle of 0.36°. Varus knees had a higher MPTA (4.26°) and TAOA (4.74°) than valgus knees (*P* < .001). MPTA and TAOA were correlated on regression analysis (r^2^ = 0.465), and all angles were statistically different between sexes.

**Conclusion:**

The angle between the TMA and floor, called TAOA, is not orthogonal in normal knees, contrary to assumptions in classical biomechanics. Knee angles vary significantly between varus and valgus cohorts, and the distinction between these cohorts should be noted when evaluating normal joint line angles.

## Introduction

In classical biomechanics, mechanical alignment of the lower extremity is defined as being achieved when the weight-bearing axis of the limb passes through the femoral head, the center of the knee joint, and the center of the talus [1,2]. Thus, for a patient to be in perfect mechanical alignment in the coronal plane, the femoral mechanical axis and the tibial mechanical axis (TMA) must be colinear and orthogonal to the floor. Furthermore, the joint line must be parallel to the floor and orthogonal to both the mechanical axes to distribute loads across the knee equally across the joint surface.

Variation of the hip-knee-ankle (HKA) axis from neutral mechanical alignment is considered to be an undesirable state [3]. Variation of the HKA from 0° is considered to be associated with uneven load distribution and to induce a varus or valgus alignment of the joint line relative to the TMA [[4], [5], [6], [7]]. A joint line that is not orthogonal to the mechanical axis is thought to predispose to injury or wear of the articular surfaces in native knees and convert normally compressive forces into damaging sheer forces [8,9].

However, true mechanical alignment is unusual in healthy human beings in bipedal stance. Studies evaluating HKA alignment in healthy patients demonstrate that significant percentages of the population are not in neutral alignment, as defined by a broadly inclusive criterion of HKA within 3° of neutral [3,[10], [11], [12]]. Furthermore, it is widely accepted that the angle between the joint line and the TMA (the medial proximal tibial angle or MPTA) is not neutral but rather averages 3° of varus. Others have brought forth the concept of constitutional varus to introduce the idea that the TMA is not orthogonal to the tibial plateau in many healthy, asymptomatic people with “bowed legs” [3].

Despite the fact that the MPTA is understood to be in varus in most patients, the joint line has also been shown to remain parallel to the floor in bipedal stance [10]. These 2 facts together challenge the assumption underlying classical biomechanics that the TMA is orthogonal to the floor. To our knowledge, the relationship between the tibial axis and the floor in bipedal stance has not been carefully reviewed. We hypothesize that in most healthy people, the TMA subtends a nonperpendicular angle with the floor to correct for the known offset between the joint line and the tibial axis. If confirmed, such a finding would redefine the frame of reference for biomechanical studies testing loads across the knee, as historically, these were constructed with the shaft of the tibia being placed perpendicular to the floor. Documenting a nonorthogonal relationship between the TMA and the loading surface of the limb has clinical implications beyond the study of biomechanics, namely for the approach to ideal alignment for knee arthroplasty.

The aim of this article is therefore to measure and quantify the radiographic alignment of the TMA relative to both 1) the weight-bearing surface of the tibia (the joint line) and 2) the floor during bipedal stance in normal knees without arthritis. Secondary outcomes include documenting the average angles subtended by the joint line to the floor, and the HKA axis of the limb, to demonstrate that the anatomy of the patients in our cohort is consistent with that of previously published articles. We further aim to identify any correlations between our findings and demographic variables. Our purpose is to challenge the assumption in classic biomechanics that the TMA is or should be considered orthogonal to the floor.

## Methods

Between January 2013 and June 2017, routine full-length radiographs were obtained by a faculty member at a large academic institution for all patients evaluated in clinic. From the pool of patients seen by this faculty, we selected for review the first 100 sequential patients with both preoperative and postoperative anterior to posterior, full-length, standing hip to ankle radiographs undergoing unilateral total knee arthroplasty (TKA), who had no evidence of joint space loss in the nonoperative knee. Demographic data were collected from the final cohort which excluded 32 patients with a contralateral TKA, traumatic or developmental deformity, or valgus ankle deformity.

**Supplemental Figure 1:**
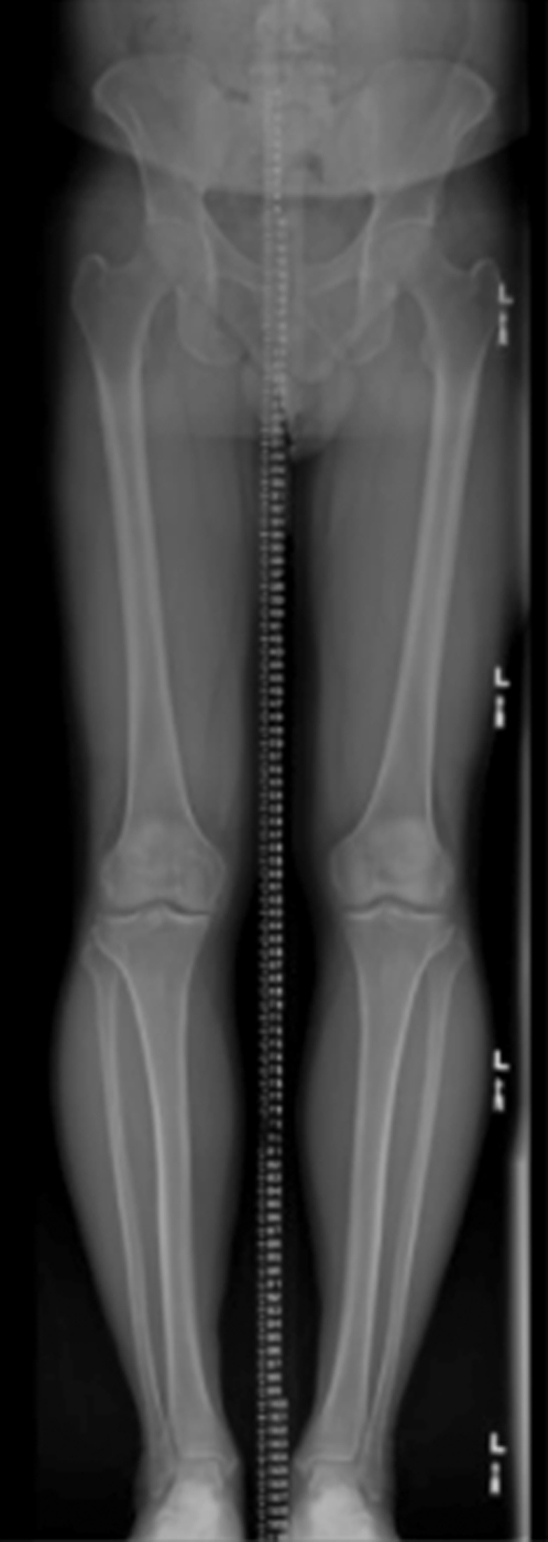
Full-length standing hip-to-ankle radiograph.

All radiographs were obtained in a standardized fashion with the patient standing upright and anteroposterior (AP) relative to the cassette with feet positioned slightly pigeon-toed within the width of the detector. Depending on the patient’s height, 3 or 4 sequential cephalad to caudal AP exposures were taken with fixed cassettes oriented parallel to the floor (from the hip, thru the knee, and down the ankle) and were automatically stitched together by the machine software (GE XR 656; GE Healthcare, Waukesha, WI) to create a full-length standing radiograph (Supp Fig. 1).

**Figure 1:**
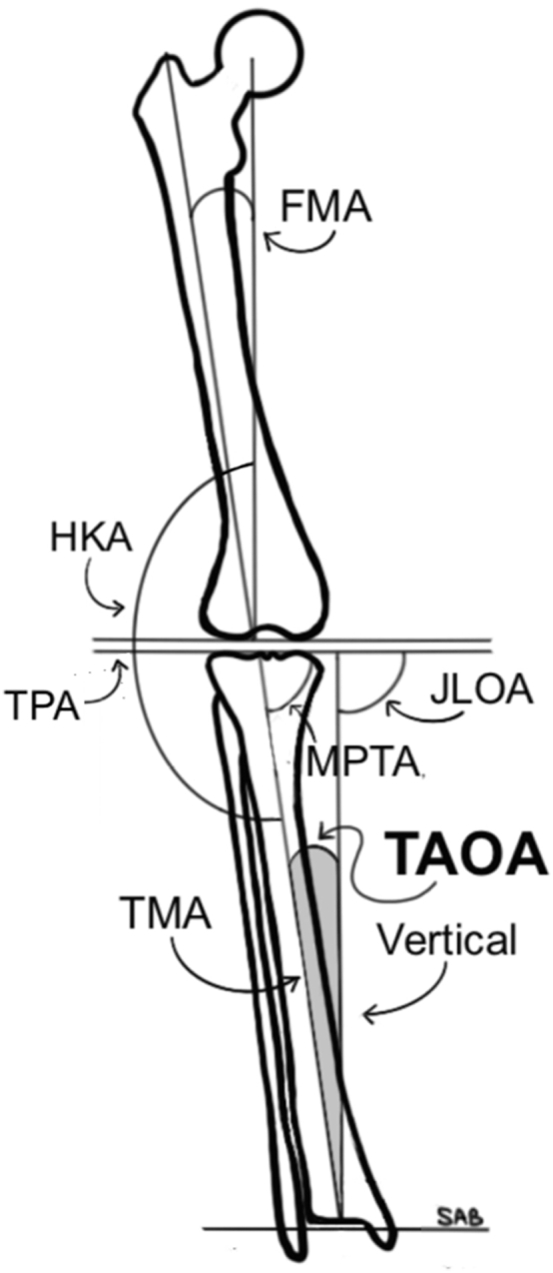
Diagram of various knee parameters and angles.

The femoral mechanical axis was defined as a line connecting the center of the femoral head and the mid-point of the knee, identified by the mid-point of the femoral condyles at the apex of the intercondylar notch. To determine the center of the femoral head, a best-fit circle was fitted around the perimeter of the femoral head. The TMA was defined as a line connecting the center of the knee and the center of the talus. The tibial plateau axis was defined by the tangent line connecting the medial and lateral tibial plateau (Fig. 1).

The HKA axis was defined as the angle between the femoral mechanical axis and the TMA, with values reported relative to the TMA [13,14]. Varus alignment was assigned a positive value, whereas valgus alignment was assigned a negative value. Neutral mechanical alignment was strictly defined at 0°. The MPTA was defined as the medial angle between the TMA and the tibial plateau axis (TPA; Fig. 1). The joint line orientation angle (JLOA) was defined as the angle between the tibial plateau axis and the floor, with positive JLOA values assigned to a medially down-sloping tibial plateau axis. The tibial axis orientation angle (TAOA), which we introduce in this article, was defined as the angle between the TMA and the floor and is reported in degrees relative to vertical. The TAOA created by a laterally positioned TMA relative to vertical (Fig. 1) was assigned a positive value.

Templating software (TraumaCad by Brainlab, Petach-Tikva, Israel) was used to obtain radiographic measurements in a blinded fashion by a single trainee under direct supervision of the senior author. Repeat measurements were obtained during a second research session, and the average of the 2 measurements was used for statistical analysis (Microsoft Excel, Redmond, WA). Intraobserver reliability was assessed using a two-way mixed effects intraclass correlation coefficient (ICC) model with absolute agreement [15], with statistical computation completed using RStudio (Version 1.3.1093; Boston, MA). ICC values between 0.75 and 0.90 indicate good reliability, and values greater than 0.90 indicate excellent reliability [15]. Significant differences in knee angle values between varus, valgus, and neutral cohorts were determined by unpaired t-test, with statistical significance set at *P* < .05. Sex-specific differences in knee angle values were determined by Chi-Square test. The associations between various knee angles and other ordinal demographic data were determined by regression analysis.

## Results

Sixty-eight of 100 patients met our study inclusion criteria. Thirty-eight females were in the cohort (Table 1), and the average age was 62.3 years (range: 30 to 83 years). Based on the values of HKA, 38 (56%) of the knees were in overall mechanical varus, 5 (7%) were in neutral alignment, and 25 (37%) of the knees were in overall mechanical valgus. For all measured knee angles, the lower bound for the 95% confidence interval of the ICC was greater than 0.9 (Table 2).

**Table 1:** Cohort demographics.

Age	62.3 (SD: 9.61)
Sex (F:M)	38:30
Height (cm)	170.8 (SD: 11.6)
Weight (kg)	89.7 (SD: 19.5)
BMI (kg/m^2^)	30.6 (SD: 5.46)

Race/Ethnicity
White/Caucasian: 42
Black/African-American: 4
Hispanic: 6
Asian/Pacific Islander: 11
Unspecified/Declined: 5

**Table 2:** Average HKA, MPTA, JLOA, TAOA (and standard deviations) for varus, valgus, and neutral alignment cohorts. Intraclass correlation coefficient (ICC) values and ICC 95% confidence intervals are reported for each angle measurement.

	n	HKA	MPTA	JLOA	TAOA
Overall	68	1.11° (3.42°)	3.06° (2.29°)	0.36° (2.23°)	2.67° (3.12°)
Valgus	25	−2.26° (2.18°)	1.24° (2.15°)	1.48° (2.08°)	−0.28° (2.20°)
Neutral	5	0° (0°)	3.00° (1.70°)	1.30° (2.22°)	1.70° (0.84°)
Varus	38	3.47° (2.12°)	4.26° (1.56°)	−0.50° (1.99°)	4.74° (2.01°)
ICC Value		0.991	0.959	0.967	0.992
ICC 95% Confidence Interval		0.986-0.994	0.939-0.972	0.950-0.978	0.988-0.994

**Figure 2:**
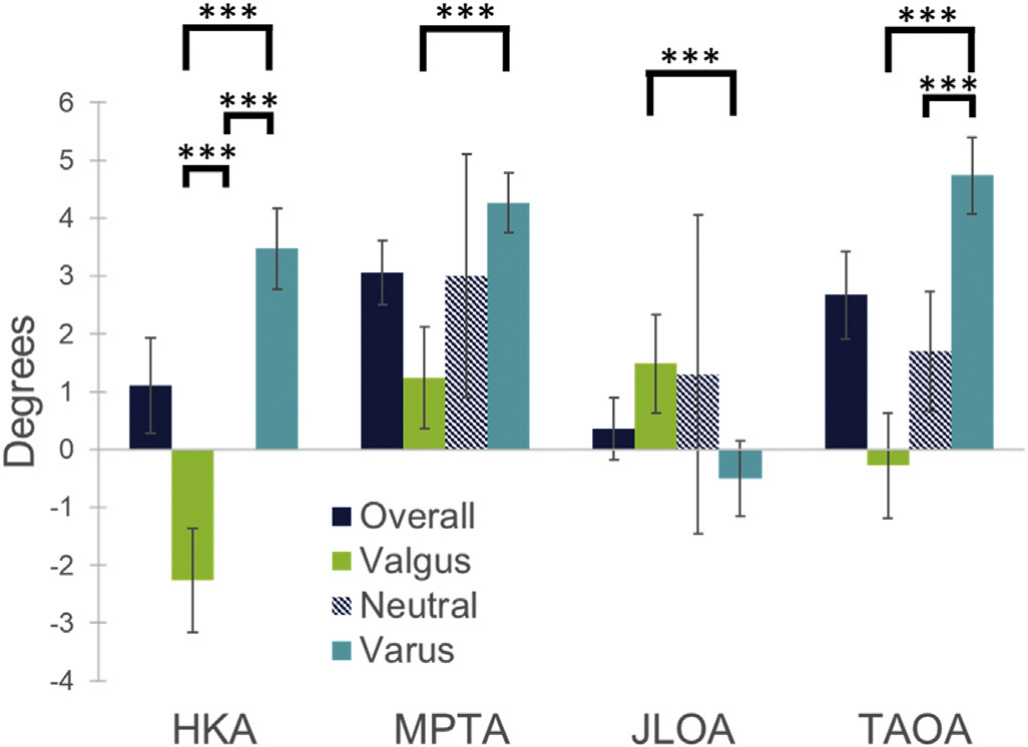
HKA, MPTA, JLOA, and TAOA highlighting statistical difference between alignment cohorts. ∗∗∗ Represents statistically significant difference between cohorts by unpaired t-test (*P* < .05).

The values for measured knee angles are noted in Table 2 and Figure 2. The TAOA, subtended by the TMA to the floor, was not orthogonal when standing, averaging 2.67° (SD: 3.13°, range: −5° to 9°) across the entire cohort. When only varus knees were considered, the TAOA increased to 4.74° (SD: 2.01°, range: 1° to 9°), which was a statistically greater angle than the valgus and neutral knee cohorts (*P* < .001 and *P* = .002, respectively). The valgus knee cohort TAOA averaged −0.28° (SD: 2.20°, range: −5° to 4°).

With respect to our secondary outcomes, the angle subtended by the tibial plateau to the TMA (MPTA) averaged 3.06° (SD: 2.31°) for the entire cohort and increased to 4.26° (SD: 1.56°, range: 1° to 6.5°) in varus knees. We found that the average value of JLOA for the entire cohort was 0.36° (SD: 2.32°), it was −0.5° (SD: 1.99°) for varus knees, and it was 1.48° (SD: 2.08°) for valgus knees, *P* < .001.

### Knee angles by demographics

There were significant differences for all knee angles when the cohort was stratified by sex (Table 3). The average HKA alignment was more varus in the male cohort (*P* = .002) with 70% of males in varus, 10% neutral, and 20% valgus, as compared to the female cohort which had 45% in varus, 5% neutral, and 50% valgus. The magnitudes of MPTA and TAOA angles were higher in males (*P* = .044 and *P* < .001, respectively). The JLOA was closer to neutral in males, with knees in the female cohort having a slight medially downsloping tibial plateau (*P* = .004).

**Table 3:** Average knee angle measurements (and standard deviations) by patient sex.

	n	HKA	MPTA	JLOA	TAOA
Female	38	−0.026° (3.45°)	2.56° (2.48°)	1.04° (2.28°)	1.43° (2.86°)
Male	30	2.55° (2.83°)	3.68° (1.86°)	−0.50° (1.87°)	4.23° (2.75°)
*P* values		0.002	0.044	0.004	0.00013

No associations with height, weight, body mass index, or age were identified for HKA, MPTA, JLOA, or TAOA in regression analysis (r^2^ < 0.01).

### Associations between MPTA, TAOA, JLOA

**Figure 3:**
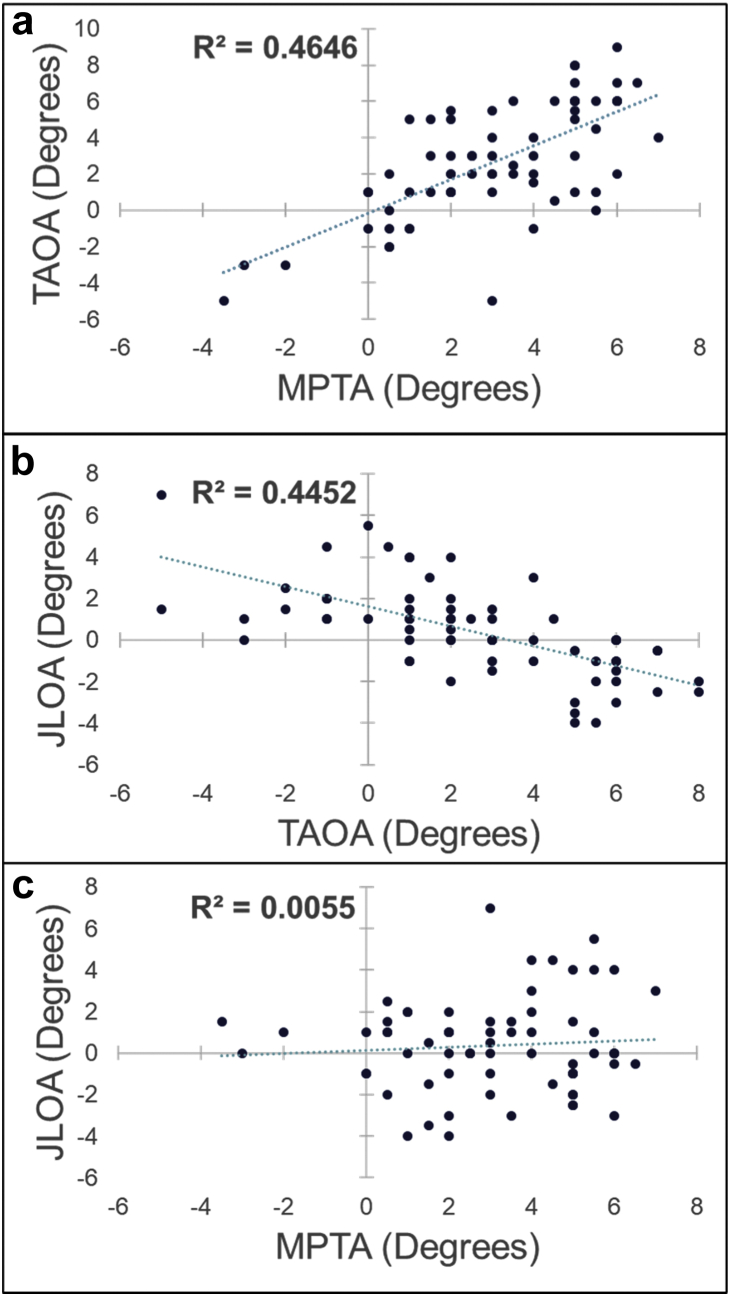
Regression analysis between knee angles: a) TAOA vs MPTA, b) JLOA vs TAOA, c) JLOA vs MPTA.

There was a positive association between the MPTA and TAOA, with r^2^ = 0.465 (Fig. 3a). While the MPTA did not correlate with the JLOA (r^2^ = 0.006, Fig. 3c), the value of TAOA was also negatively associated with JLOA (r^2^ = 0.445; Fig. 3b).

**Figure 4:**
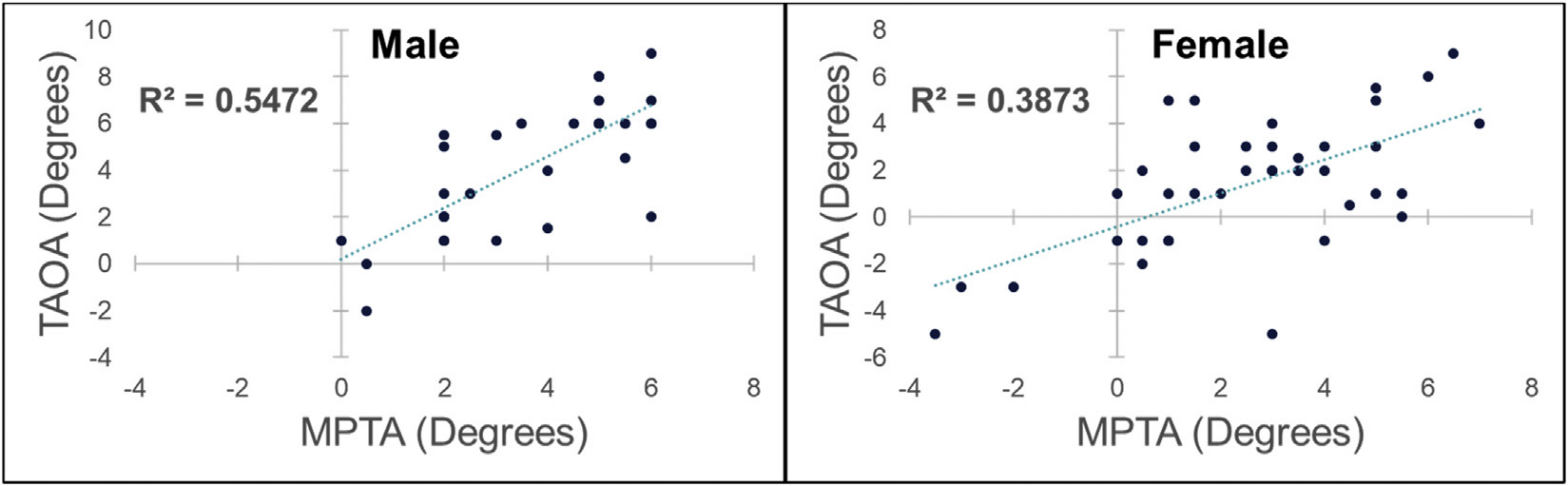
TAOA vs MPTA stratified by sex.

When the study group was separated into male and female cohorts, the positive association between MPTA and TAOA was retained (r^2^ = 0.547 and 0.387, respectively; Fig. 4).

## Discussion

The major finding from our evaluation of full-length, standing AP radiographs in a cohort of asymptomatic knees is that the angle subtended between the TMA and the floor, which we name the TAOA, deviates on average over 2.5° from the orthogonal. We also analyze varus and valgus knees separately to demonstrate that in varus knees, the TAOA is actually much higher, averaging 4.7° in normal knees. Furthermore, a key finding in this study was the wide distribution noted in the TAOA, ranging from −5° to 9° with a standard deviation of 3.1°. This is contrary to the long-standing assumption underlying mechanical studies of the knee that the TAOA is in neutral alignment. This degree of variance was previously not well documented and makes many historical biomechanical studies of the knee, in which the tibia is placed in neutral alignment, difficult to apply clinically.

Our anatomic data are in agreement with prior published findings, demonstrating that the JLOA is neutral to the floor and that the MPTA is approximately 3° varus [3,16,17]. We further show how the MPTA varies more significantly between varus and valgus knees than previously appreciated. In the valgus knee cohort, for example, the MPTA is nearly orthogonal to the TMA. Not surprisingly, the TAOA is therefore also nearly orthogonal to the floor in valgus knees. Conversely, the varus knee cohort demonstrates much greater values of the MPTA, averaging 4.26°, which is larger than the 3° commonly cited as the maximum upper normal variance for the MPTA [3]. If we were to define the acceptable range of varus MPTA as one standard deviation from average, the upper limit of normal would be 5.8°. In this subgroup of varus knees, we observed a compensatory increase in the TAOA that realigns the tibial plateau parallel to the floor in most cases. However, it is worth noting that the TAOA is not always equal to the MPTA and does not always fully return the JLOA to neutral.

A relationship between the magnitudes of MPTA and TAOA is similarly observed when the cohort is stratified by sex. The male cohort in our study demonstrates better overall varus alignment, with greater magnitudes of MPTA and compensatory TAOA, than the female cohort which on average has a more neutral mechanical alignment.

Our data demonstrate an association between MPTA and TAOA, and JLOA with TAOA, but the magnitude of MPTA appears not to directly correlate with the JLOA. However, while the JLOA has a limited range and is expected to remain within 2° of neutral in over 60% of patients, the TAOA and MPTA have wider ranges with only approximately 30% of patients expected to fall within 2° of neutral. Based on this observation, we argue that joint line orientation relative to the floor is the anatomic variable that is most preserved in nature. Furthermore, a neutral joint line in vivo can be achieved by recreating the anatomic TAOA to compensate for a varus MPTA.

The data from this cohort mirror findings reported by other authors that, on average, the tibial joint line is in approximately 3° of varus relative to the TMA [3,16]. However, we are among the first to emphasize that there exists a marked variation in the MPTA between varus and valgus knees and that, on average, the varus knee has a much higher MPTA than one sees in cohorts that include valgus joints. This variability represents the normal range of joint line angulation and is unlikely to be a cause of early degenerative wear, as we would expect a much higher incidence and prevalence of osteoarthritis in the general population if that were the case. Furthermore, even in Asian population studies [11,[18], [19], [20]] with a relatively high degree of varus knee angulation, several authors have failed to find a correlation between a varus MPTA and osteoarthritis [16], or a contribution of a varus mechanical axis to the development of osteoarthritis [[21], [22], [23], [24], [25]].

We chose to define mechanical alignment as HKA equal to 0°, a definition that is more rigorous than the range used in other studies, and found that true mechanical alignment is an exception for in vivo knees. We acknowledge that using an alignment of ±3° is appropriate for use in postoperative evaluation studies to account for measurement error between radiographs taken at different times of the same individual. However, our study analyzes the naturally occurring distribution of varus, neutral, and valgus alignment, and we see no reason to consider a knee “neutral” when it is measurably not in neutral alignment. To further demonstrate the generalizability of our cohort, we performed a subanalysis in which we stratified our cohort based on the ±3° definition and found a similar overall distribution of varus, neutral, and valgus knees as seen in other studies [3,10] (Supp. Table 1).

The strength of our study is in analyzing radiographic angles with full-length, standing films, which evaluates the in vivo biomechanics during bipedal stance. In addition, our cohort size is fairly robust for a pilot analysis of the TAOA, and we have demonstrated the generalizability of our results with respect to previously published analyses on nonarthritic knee angles in Western populations, suggesting that our data set is representative of the greater population in the United States [3,10,12,16,17,26,27].

Our study is limited by the retrospective nature of its analysis, and we acknowledge that using manual techniques to measure angles on two-dimensional radiographs is susceptible to error because of rotation, projection, and variations in manual measurements. We attempted to address this by repeating the study in a blinded fashion and selecting anatomic landmarks that are easy to reproduce. As we saw negligible variation between measurements, with ICC values greater than 0.90 indicating excellent intraobserver reliability [15], we believe our data correctly report the anatomy of our cohort. There are several factors that may affect the position of the normal contralateral limb, in particular the height of the patient and any flexion contractures of the diseased knee. In the group that we reviewed, the variation we measured on the healthy knee was limited, suggesting that the impact of the diseased limb on overall alignment of the healthy knee is unlikely to have affected our results. It should be noted that this study assesses the knee in bipedal stance, a static position, and that the angle measurements reported may vary through the gait cycle. Further study of dynamic changes in JLOA and TAOA are warranted and may demonstrate wider variation with movement, particularly as the foot moves closer to midline during heel-strike.

Our study evaluates the contralateral, nonoperative knee in patients who have undergone total knee replacement, and it may be argued that this population represents abnormal anatomy that is predisposed to developing osteoarthritis. Our cohort also may not reflect the symptomatic status of the nonoperative knee. However, the fact that the values we report for HKA, MPTA, and JLOA in the nonoperative knee are nearly identical to those previously studied and accepted as normal for the human population makes these concerns less applicable for our cohort [3,10,12,16,17,26,27].

## Conclusions

In summary, we documented that in bipedal stance, the TMA is not vertical but rather deviates by 2.7° on average relative to a line drawn orthogonally to the floor (TAOA). Differently stated, an approximately 3° offset of the tibia should be considered the average in healthy knees, not the upper limit of normal.

Furthermore, a combined analysis of varus and valgus knees grossly underestimates the true variation in proximal tibial geometry. The varus knees in this series demonstrated an average TAOA of 4.7° (SD: 2.01°, range: 1° to 9°) while valgus knees averaged a TAOA of −0.28° (SD: 2.20°, range: −5° to 4°). We further corroborate other studies that found the joint line to be parallel to the floor in most patients. Our findings challenge the foundational idea in classical mechanical alignment models that the tibia should be positioned and loaded orthogonally to the floor in an effort to reproduce normal anatomy. Future biomechanical studies looking to determine load distribution across the knee must position the tibia correctly if they are to replicate physiologic in vivo loading. Furthermore, to truly understand the relationship of a TKA to the mechanical axis of the lower extremity, weight-bearing (and, ideally, full length) radiographs must be obtained to assess the MPTA, JLOA, and TAOA.

## Conflicts of interest

The authors declare the following financial interests/personal relationships which may be considered as potential competing interests: Stefano Bini, MD, declares stock or stock options in Cloudmedix, InSilico Trials, Sira Medical, and CaptureProof and receives royalties from Stryker. Stefano Bini, MD, is the Digital Health and Social Media Committee Chair of the American Association of Hip and Knee Surgeons and serves on the Editorial Board for Journal of Arthroplasty and Arthroplasty Today. Erik Hansen, MD, is a book editor for Heraeus and receives royalties from Corin.

## Figures and Tables

**Supplemental Table 1:** Average knee angle values (and standard deviations) for alignments cohorts using definitions of varus alignment as HKA ≥3° and valgus alignment HKA ≤ −3°.

	n	HKA	MPTA	JLOA	TAOA
Overall	68	1.11° (3.42°)	3.06° (2.29°)	0.36° (2.23°)	2.67° (3.12°)
Valgus	5 (7.4%)	−5.90° (2.27°)	−0.10° (2.97°)	2.50° (2.64°)	−2.60° (2.61°)
Neutral	47 (69.1%)	0.35° (1.74°)	2.69° (1.94°)	0.55° (2.17°)	2.12° (2.33°)
Varus	16 (23.5%)	5.53° (1.56°)	5.12° (1.07°)	−0.88° (1.65°)	5.94° (1.87°)

## Appendix A Supplementary data

Conflict of Interest Statement for Chung

Conflict of Interest Statement for Hansen

Conflict of Interest Statement for Bini

Conflict of Interest Statement for Wu
