# Supplementary material for: Tibial Mechanical Axis Is Nonorthogonal to the Floor in Varus Knee Alignment
Source: Arthroplast Today. 2021 Apr 15;8:237–42. doi: 10.1016/j.artd.2021.03.009 (PMC8079331; doi:10.1016/j.artd.2021.03.009)
Supplement: Conflict of Interest Statement for Bini [file mmc3.pdf]

INDIVIDUAL CONFLICT OF INTEREST STATEMENT

American Association of Hip and Knee Surgeons

(Adopted from the American Academy of Orthopaedic Surgeons disclosure statement)

The following form **must be filled out completely and submitted by each author (example, 6 authors, 6 forms). All items require a response. If there is no relevant disclosure for a given item, enter "*None*."**

Tibial Mechanical Axis is Non-orthogonal to the Floor in Varus Knee Alignment

---

Manuscript Title

1. Royalties from a company or supplier (The following conflicts were disclosed)

Stryker

2. Speakers bureau/paid presentations for a company or supplier (The following conflicts were disclosed)

3A. Paid employee for a company or supplier (The following conflicts were disclosed)

3B. Paid consultant for a company or supplier (The following conflicts were disclosed)

3C. Unpaid consultants for a company or supplier (The following conflicts were disclosed)

4. Stock or stock options in a company or supplier (The following conflicts were disclosed)

Cloudmedix, InSilico Trials, Sira Medical, CaptureProof

5. Research support from a company or supplier as a Principal Investigator (The following conflicts were disclosed)

6. Other financial or material support from a company or supplier (The following conflicts were disclosed)

7. Royalties, financial or material support from publishers (The following conflicts were disclosed)

8. Medical/Orthopaedic publications editorial/governing board (The following conflicts were disclosed)

Journal Of Arthroplasty, Arthroplasty Today

9. Board member/committee appointments for a society (The following conflicts were disclosed)

American Association of Hip and Knee Surgeons

Each author must sign AND print or type his/her name, date and submit a separate form

In addition, one BLINDED Conflict of Interest form (no author names used) should be submitted per manuscript with all author disclosures.

Stefano Bini [signature] 10.27.20

Author Name (Print or Type) Author Signature Date
